# Supplementary material for: Structural basis of measles virus polymerase inhibition by nonnucleoside inhibitor ERDRP-0519
Source: Nat Commun. 2025 Oct 13;16:9061. doi: 10.1038/s41467-025-64128-0 (PMC12518664; doi:10.1038/s41467-025-64128-0)
Supplement: Supplementary file 1 — Supplementary Information [file 41467_2025_64128_MOESM1_ESM.pdf]

Supplementary Information for

Structural Basis of Measles Virus Polymerase inhibition by nonnucleoside inhibitor ERDRP-0519

Dong Wang[1†], Fan Bu[1,2†], Ge Yang[1], Bin Liu[1*]

[1]Section of Transcription & Gene Regulation, The Hormel Institute, University of Minnesota, Austin, MN, USA

[2]Department of Pharmacology, University of Minnesota Medical School, Minneapolis, Minnesota, USA

[†]These authors contributed equally.

*Correspondence to: liu00794@umn.edu (B.L.)

Supplementary Fig. 1-3

Supplementary Table 1-2

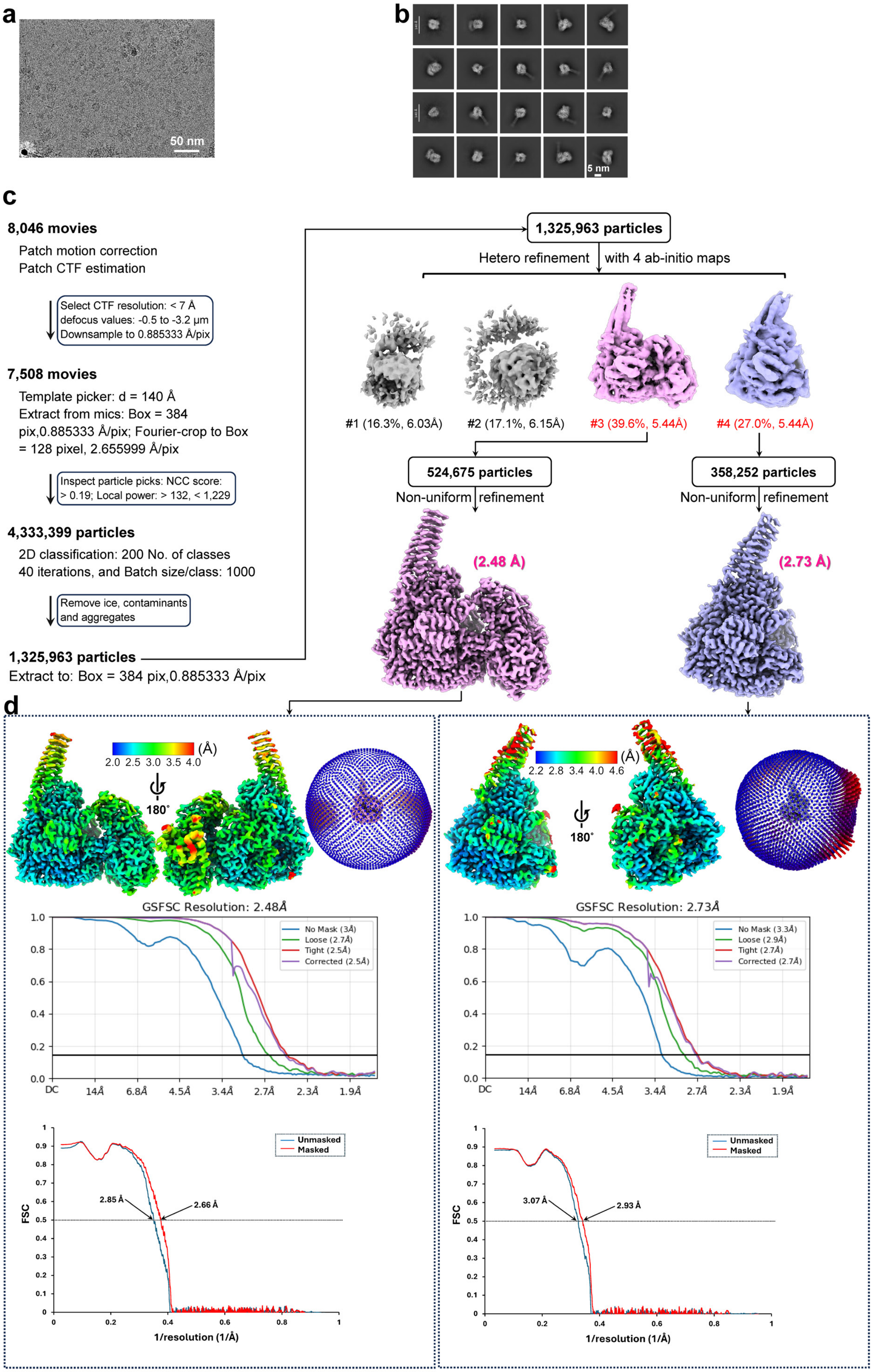

a
50 nm
b
5 nm
c
8,046 movies
Patch motion correction
Patch CTF estimation
Select CTF resolution: < 7 Å
defocus values: -0.5 to -3.2 µm
Downsample to 0.885333 Å/pix
7,508 movies
Template picker: d = 140 Å
Extract from mics: Box = 384 pix,0.885333 Å/pix; Fourier-crop to Box = 128 pixel, 2.655999 Å/pix
Inspect particle picks: NCC score: > 0.19; Local power: > 132, < 1,229
4,333,399 particles
2D classification: 200 No. of classes
40 iterations, and Batch size/class: 1000
Remove ice, contaminants and aggregates
1,325,963 particles
Extract to: Box = 384 pix,0.885333 Å/pix
1,325,963 particles
Hetero refinement with 4 ab-initio maps
#1 (16.3%, 6.03Å)
#2 (17.1%, 6.15Å)
#3 (39.6%, 5.44Å)
#4 (27.0%, 5.44Å)
524,675 particles
358,252 particles
Non-uniform refinement
Non-uniform refinement
(2.48 Å)
(2.73 Å)
d
(Å)
2.0 2.5 3.0 3.5 4.0
180˚
(Å)
2.2 2.8 3.4 4.0 4.6
180˚
GSFSC Resolution: 2.48Å
No Mask (3Å)
Loose (2.7Å)
Tight (2.5Å)
Corrected (2.5Å)
GSFSC Resolution: 2.73Å
No Mask (3.3Å)
Loose (2.9Å)
Tight (2.7Å)
Corrected (2.7Å)
DC 14Å 6.8Å 4.5Å 3.4Å 2.7Å 2.3Å 1.9Å
Unmasked
Masked
2.85 Å
2.66 Å
3.07 Å
2.93 Å
FSC
1/resolution (1/Å)

Supplementary Fig. 1: Cryo-EM data processing workflow.

a-b, The representative raw cryo-EM image and 2D classes are presented with scale bars. **c,** Detailed cryo-EM data processing procedure of the MeV polymerase complex-ERDRP-0519 is outlined. 3D refinement using all the particles in the good 3D classes generated the 2.73 Å and 2.48 Å resolution maps for MeV L_{core}-P and L_{full}-P-C complexes, respectively. **d,** Local resolution illustrations, angular distribution plot, half-map FSC curves, and model-to-map FSCs are enclosed in the dashed black box.

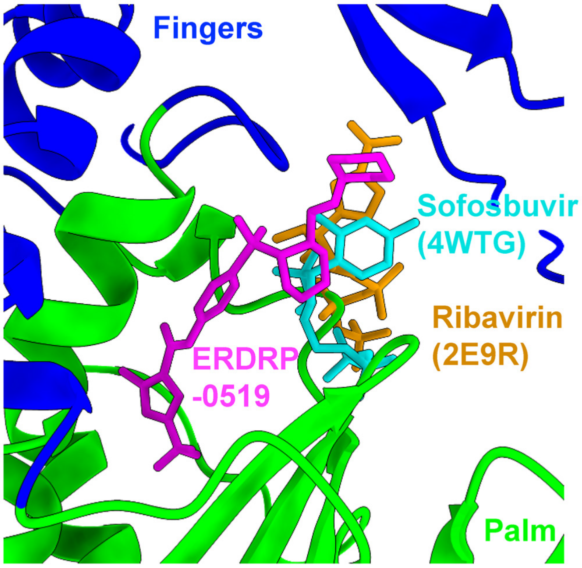

Supplementary Fig. 2: Superimposition of different compounds bound to polymerases. HCV NS5B–Sofosbuvir (PDB: 4WTG) and FMDV RdRp–Ribavirin (PDB: 2E9R) are superimposed onto MeV L- ERDRP-0519. The color scheme of MeV L RdRp domain is the same as in Fig. 1d. Drug molecules are colored as follows: Sofosbuvir (cyan), Ribavirin (orange), and ERDRP-0519 (purple).

```
                     Motif A                     Motif B             Motif C      Motif D
                   661        671   731         741         751  771         811
MeV/Edm            TTDLKKYCLN WRYET FIKYPMGGIE GYCQKLWTIS T    QGDNQTIAVT LHDIGHHLKA
MeV/B3             TTDLKKYCLN WRYET FIKYPMGGIE GYCQKLWTIS T    QGDNQTIAVT LHDIGHHLKA
MeV/D4             TTDLKKYCLN WRYET FIKYPMGGIE GYCQKLWTIS T    QGDNQTIAVT LHDIGHHLKA
MeV/D8             TTDLKKYCLN WRYET FIKYPMGGIE GYCQKLWTIS T    QGDNQTIAVT LHDIGHHLKA
MeV/H1             TTDLKKYCLN WRYET FIKYPMGGIE GYCQKLWTIS T    QGDNQTIAVT LHDIGHHLKA
                   ********** ***** ********** ********** *    ********** **********
```

Supplementary Fig. 3: Sequence alignment of the RdRp from MeV isolates. Edmonston-A (GenBank: K01711.1); B3 (GenBank: XDZ84916.2); D4 (GenBank: AVA07214.1); D8 (GenBank: WZD64797.1); H1 (GenBank: AVA07220.1).

Supplementary Table 1. Cryo-EM data collection, refinement, and validation statistics.

	MeV L-P-ERDRP-0519 (EMD-70313) (PDB 9OCF)	MeV L-P-C-ERDRP-0519 (EMD-70312) (PDB 9OCE)
**Data collection and processing**		
Magnification	130,000	130,000
Voltage (kV)	300	300
Electron exposure (e–/Å^2)	50.0	50.0
Defocus range (μm)	-1.0 ~ -2.0	-1.0 ~ -2.0
Pixel size (Å)	0.664	0.664
Symmetry imposed	C1	C1
Initial particle images (no.)	1,325,963	1,325,963
Final particle images (no.)	358,252	524,675
Map resolution (Å)	2.73	2.48
FSC threshold	0.143	0.143
Map resolution range (Å)	2.2-4.6	2.0-4.0
**Refinement**		
Initial model used (PDB code)	9DUS	9DUT
Model resolution (Å)	2.93	2.66
FSC threshold	0.5	0.5
Map sharpening *B* factor (Å^2)	-88.7	-78.2
Model composition		
Non-hydrogen atoms	12449	20404
Protein residues	1558	2558
Ligands	1	1
B factors (Å^2)		
Protein	124.94	134.17
Ligand	105.86	93.36
R.m.s. deviations		
Bond lengths (Å)	0.003	0.005
Bond angles (°)	0.727	0.902
**Validation**		
MolProbity score	1.15	1.27
Clashscore	2.36	2.62
Poor rotamers (%)	0.00	0.00
Ramachandran plot		
Favored (%)	97.26	96.57
Allowed (%)	2.74	3.43
Disallowed (%)	0.00	0.00

Supplementary Table 2. Summary of the primers used in the mutagenesis.

Primer name	Primer sequence (5′-3′)
L-D773A Forward	CAAGGGGCCAATCAGACCATAGCCGTAAC
L-D773A Reverse	TGGTCTGATTGGCCCCTTGCACTAACGAAGC
L-L664A Forward	GACTGATGCCAAGAAGTACTGCCTTAATTG
L-L664A Reverse	AGTACTTCTTGGCATCAGTCGTGATAAATGC
L-W671F Forward	CCTTAATTTCAGATATGAGACCATCAGCTTG
L-W671F Reverse	TCTCATATCTGAAATTAAGGCAGTACTTCTTG
L-T751A Forward	CATCAGCGCCATTCCCTATCTATACCTGGC
L-T751A Reverse	GATAGGGAATGGCGCTGATGGTCCACAGCTTC
L-H816L Forward	TATTGGCCTTCACCTCAAGGCAAATGAGAC
L-H816L Reverse	CCTTGAGGTGAAGGCCAATATCATGTAGCC
