# Supplementary material for: Structural basis of measles virus polymerase inhibition by nonnucleoside inhibitor ERDRP-0519
Source: Nat Commun. 2025 Oct 13;16:9061. doi: 10.1038/s41467-025-64128-0 (PMC12518664; doi:10.1038/s41467-025-64128-0)
Supplement: Supplementary file 2 — Reporting Summary [file 41467_2025_64128_MOESM2_ESM.pdf]

nature portfolio

Corresponding author(s): Bin Liu

Last updated by author(s): Aug 25, 2025

# Reporting Summary

Nature Portfolio wishes to improve the reproducibility of the work that we publish. This form provides structure for consistency and transparency in reporting. For further information on Nature Portfolio policies, see our Editorial Policies and the Editorial Policy Checklist.

## Statistics

For all statistical analyses, confirm that the following items are present in the figure legend, table legend, main text, or Methods section.

| n/a | Confirmed | |
|---|---|---|
| ☐ | ☒ | The exact sample size (*n*) for each experimental group/condition, given as a discrete number and unit of measurement |
| ☐ | ☒ | A statement on whether measurements were taken from distinct samples or whether the same sample was measured repeatedly |
| ☐ | ☒ | The statistical test(s) used AND whether they are one- or two-sided<br>*Only common tests should be described solely by name; describe more complex techniques in the Methods section.* |
| ☐ | ☒ | A description of all covariates tested |
| ☐ | ☒ | A description of any assumptions or corrections, such as tests of normality and adjustment for multiple comparisons |
| ☐ | ☒ | A full description of the statistical parameters including central tendency (e.g. means) or other basic estimates (e.g. regression coefficient) AND variation (e.g. standard deviation) or associated estimates of uncertainty (e.g. confidence intervals) |
| ☐ | ☒ | For null hypothesis testing, the test statistic (e.g. *F*, *t*, *r*) with confidence intervals, effect sizes, degrees of freedom and *P* value noted<br>*Give P values as exact values whenever suitable.* |
| ☒ | ☐ | For Bayesian analysis, information on the choice of priors and Markov chain Monte Carlo settings |
| ☒ | ☐ | For hierarchical and complex designs, identification of the appropriate level for tests and full reporting of outcomes |
| ☒ | ☐ | Estimates of effect sizes (e.g. Cohen's *d*, Pearson's *r*), indicating how they were calculated |

*Our web collection on statistics for biologists contains articles on many of the points above.*

## Software and code

Policy information about availability of computer code

| | |
|---|---|
| Data collection | EPU version 2.5 |
| Data analysis | MotionCor2; CTFFIND-4.1.13; cryoSPARC v4.5.1; Chimera v1.16; Molprobity, Phenix-1.16; Coot-0.8.9; UCSF ChimeraX 1.7; Clustal Omega web server (https://www.ebi.ac.uk/jdispatcher/msa/clustalo), ESPript 3.0 web server (https://espript.ibcp.fr/ESPript/ESPript/index.php). |

For manuscripts utilizing custom algorithms or software that are central to the research but not yet described in published literature, software must be made available to editors and reviewers. We strongly encourage code deposition in a community repository (e.g. GitHub). See the Nature Portfolio guidelines for submitting code & software for further information.

## Data

Policy information about availability of data

All manuscripts must include a data availability statement. This statement should provide the following information, where applicable:

- Accession codes, unique identifiers, or web links for publicly available datasets
- A description of any restrictions on data availability
- For clinical datasets or third party data, please ensure that the statement adheres to our policy

The atomic coordinate of MeV L-P-ERDRP-0519 complex and L-P-C-ERDRP-0519 complex have been deposited in PDB with accession number 9OCF [http://doi.org/10.2210/pdb9OCF/pdb] and 9OCE [https://doi.org/10.2210/pdb9OCE/pdb], respectively. The cryo-EM density map of MeV L-P-ERDRP-0519 complex and L-P-C-ERDRP-0519 complex have been deposited in the Electron Microscopy Data Bank with accession number EMD-70313 [https://www.ebi.ac.uk/pdbe/entry/emdb/EMD-70313] and EMD-70312 [https://www.ebi.ac.uk/pdbe/entry/emdb/EMD-70312], respectively. The relevant published structural data are represented by

the following PDB accession number, including NiV L-P-RNA (PDB: 9GJU) [https://doi.org/10.2210/pdb9GJU/pdb], HCV NS5B–Sofosbuvir (PDB: 4WTG) [https://doi.org/10.2210/pdb4WTG/pdb] and FMDV RdRp–Ribavirin (PDB: 2E9R) [https://doi.org/10.2210/pdb2E9R/pdb].

## Research involving human participants, their data, or biological material

Policy information about studies with human participants or human data. See also policy information about sex, gender (identity/presentation), and sexual orientation and race, ethnicity and racism.

| | |
|---|---|
| Reporting on sex and gender | N/A |
| Reporting on race, ethnicity, or other socially relevant groupings | N/A |
| Population characteristics | N/A |
| Recruitment | N/A |
| Ethics oversight | N/A |

Note that full information on the approval of the study protocol must also be provided in the manuscript.

# Field-specific reporting

Please select the one below that is the best fit for your research. If you are not sure, read the appropriate sections before making your selection.

☒ Life sciences ☐ Behavioural & social sciences ☐ Ecological, evolutionary & environmental sciences

For a reference copy of the document with all sections, see nature.com/documents/nr-reporting-summary-flat.pdf

# Life sciences study design

All studies must disclose on these points even when the disclosure is negative.

| | |
|---|---|
| Sample size | Sufficient cryo-EM data were collected to achieve adequate map resolutions for model building. |
| Data exclusions | No data were excluded from analyses. |
| Replication | Sample preparation-related experiments including protein purification and enzymatic assays were reproduced at least twice independently. All attempts at replication were successful. |
| Randomization | Randomization was not relevant to our study because our study did not involve the allocation of samples/organisms/participants into experimental groups. |
| Blinding | Investigators were not blinded to group allocation because group allocation was not involved in our study. Investigators were not blinded during data collection because the data being collected were quantitative in nature and were not prone to subjective interpretation. |

# Reporting for specific materials, systems and methods

We require information from authors about some types of materials, experimental systems and methods used in many studies. Here, indicate whether each material, system or method listed is relevant to your study. If you are not sure if a list item applies to your research, read the appropriate section before selecting a response.

### Materials & experimental systems

| n/a | Involved in the study | |
|---|---|---|
| ☐ | ☒ | Antibodies |
| ☐ | ☒ | Eukaryotic cell lines |
| ☒ | ☐ | Palaeontology and archaeology |
| ☒ | ☐ | Animals and other organisms |
| ☒ | ☐ | Clinical data |
| ☒ | ☐ | Dual use research of concern |
| ☒ | ☐ | Plants |

### Methods

| n/a | Involved in the study | |
|---|---|---|
| ☒ | ☐ | ChIP-seq |
| ☒ | ☐ | Flow cytometry |
| ☒ | ☐ | MRI-based neuroimaging |

## Antibodies

| | |
|---|---|
| Antibodies used | Rabbit anti-HA (1:2000, Cell Signaling Technology, C29F4), mouse anti-His (1:3000, Invitrogen, His.H8, MA1-21315), and mouse anti-Strep (1:1000, IBA, 2-1507-001). Detection of the primary antibodies was carried out using HRP-conjugated secondary antibodies |

| | |
|---|---|
| | (Cell Signaling Technology), including goat anti-rabbit (7074S) and rabbit anti-mouse (58802S), each at 1:5000. |
| Validation | All antibodies used in this study were validated by the manufacturer.<br>Rabbit anti-HA-tag monoclonal antibody https://www.cellsignal.com/products/primary-antibodies/ha-tag-c29f4-rabbit-mab/3724?srsltid=AfmBOoovlaiF-Yn7exnZWGHRV8kSpiPw9LQglEy4rg0sX5pM4LFr1j3Z<br>Mouse anti-6X His-tag monoclonal antibody https://www.thermofisher.com/antibody/product/6x-His-Tag-Antibody-clone-HIS-H8-Monoclonal/MA1-21315<br>Mouse anti-strep-tag monoclonal antibody https://www.iba-lifesciences.com/strepmab-classic/2-1507-001<br>Goat anti-rabbit HRP-conjugated secondary antibody https://www.cellsignal.com/products/secondary-antibodies/anti-rabbit-igg-hrp-linked-antibody/7074<br>Rabbit anti-mouse HRP-conjugated secondary antibody https://www.cellsignal.com/products/secondary-antibodies/rabbit-anti-mouse-igg-light-chain-specific-d3v2a-mab-hrp-conjugate/58802 |

## Eukaryotic cell lines

Policy information about cell lines and Sex and Gender in Research

| | |
|---|---|
| Cell line source(s) | Spodoptera frugiperda(sf9) cells(ThermorFisher), Trichoplusia ni(Tni) cells(Expression Systems), HEK293T cells(ATCC) |
| Authentication | The cell line was purchased from ThermoFisher, Expression Systems and ATCC was not authenticated |
| Mycoplasma contamination | Mycoplasma testing revealed no contamination. |
| Commonly misidentified lines (See ICLAC register) | No commonly misidentified cell lines were used |

## Plants

| | |
|---|---|
| Seed stocks | N/A |
| Novel plant genotypes | N/A |
| Authentication | N/A |
